# Melanoma in Pregnancy—Diagnosis, Treatment, and Consequences for Fetal Development and the Maintenance of Pregnancy

**DOI:** 10.3390/cancers16122173

**Published:** 2024-06-07

**Authors:** Patrycja Pelczar, Pola Kosteczko, Ewelina Wieczorek, Maciej Kwieciński, Aleksandra Kozłowska, Paulina Gil-Kulik

**Affiliations:** 1 Student Scientific Society of Clinical Genetics, Medical University of Lublin, 11 Radziwillowska Str., 20-080 Lublin, Poland; 59789@student.umlub.pl (P.P.); pola.kosteczko@wp.pl (P.K.); 55937@student.umlub.pl (E.W.); maciek@wfk.net.pl (M.K.); 2 Department of Radiotherapy, Medical University of Lublin, 13 Radziwillowska Str., 20-080 Lublin, Poland; aleksandra.kozlowska@umlub.pl; 3 Department of Clinical Genetics, Medical University of Lublin, 11 Radziwillowska Str., 20-080 Lublin, Poland

**Keywords:** pregnancy, melanoma, treatment, diagnosis, fetus

## Abstract

**Simple Summary:**

Pregnancy-associated cancers (PACs) represent a significant clinical problem, due to their possibly delayed diagnosis, limitations of the usage of diagnostic and therapeutic methods, and often the necessity to postpone the administration of optimal treatment to the mother until after delivery to protect the health of the fetus. The purpose of this review is to summarize information on the epidemiology, proper diagnosis, and effective treatment of cutaneous malignant melanoma (CMM) during pregnancy. Moreover, our aim is to point out that there are still many topics related to pregnancy-associated melanoma (PAM) that require further research. The following article can help clinicians systematize the updated facts from various studies and case-reports as well as advise them that an interdisciplinary approach combined with broad knowledge from different fields of medicine is crucial in the treatment of PAM.

**Abstract:**

Cutaneous malignant melanoma is one of the most common neoplasms among pregnancy-associated cancers (PACs). Risk factors include excessive exposure to ultraviolet radiation, the presence of benign and dysplastic nevi, and a patient or family history of melanoma. Self-examination and careful inspection of nevi are crucial, especially in the context of their progression over time. Physiological changes that occur during pregnancy, such as the darkening and enlargement of the nevi, delay the diagnosis of CMM. In the fetus, metastases are very rare, and if they do occur, they concern the placenta or fetal tissues. The choice of treatment is influenced by the cancer stage, symptoms, the time of termination of pregnancy, and the patient’s decision. Essential procedures which are safe for the fetus are diagnostic biopsy, ultrasound, and the therapeutic excision of the lesion and the affected lymph nodes. Other imaging methods can be used with a safe radiation dose limit of 100 mGy. Immunotherapy and targeted treatments must be carefully considered, because of their possible adverse effects on the fetus. An interdisciplinary approach to the problem of melanoma during pregnancy is necessary, involving doctors of various specialties.

## 1. Introduction

Cutaneous melanoma is a malignant neoplasm that originates in melanocytes situated in the basal layer of the epidermis. Melanocytes are responsible for producing the dark pigment known as melanin, which imparts color to the skin. The uncontrolled growth of these cells characterizes melanoma [1,2,3,4]. However, since melanocytes are also present in the eyes, ears, gastrointestinal tract, genitalia, urinary system, and meninges, cases of melanoma may also occur in these locations [3,4]. There are several clinical subtypes of melanoma, each characterized by distinct presentations, demographics, and molecular profiles. Among these, superficial spreading melanoma (SSM) is the most prevalent type within cutaneous melanoma [2].

Melanoma stands as the third most common cutaneous malignancy, trailing behind basal-cell and squamous-cell carcinomas [1,3]. However, melanoma contributes to approximately three-quarters of all deaths attributed to skin cancer [2,3].

The incidence of cutaneous melanoma continues to rise steadily. Nevertheless, there are discrepancies in the frequency of occurrence depending on geographical location. The fair-skinned phenotype, especially in regions with intense sun exposure, experiences the highest incidence of cutaneous melanoma [1,2,3,4,5]. The highest incidence of melanoma is observed in regions such as New Zealand, Australia, and Europe [1,6].

In the context of gender in the case of melanoma, both the incidence and mortality rates are higher among men than women [6]. However, females in the age range of 20–24 are at a higher risk of being diagnosed with malignant melanoma compared to males. This is probably because young women tend to engage in behaviors that pose a higher risk of developing melanoma, such as sunbathing, driven by socially influenced aesthetic preferences [1,3,5]. Around 35% of women diagnosed with melanoma are of reproductive age, rendering it one of the most commonly detected cancers in pregnant women. The precise occurrence of malignant melanoma during pregnancy remains unknown. According to Anderson et al. and their Swedish population-based studies, melanoma is the most common malignancy reported during pregnancy [7].

As a general estimate, in the INCIP register until August 2019, it is believed that melanoma constitutes around 5% of all malignant tumors that develop during pregnancy. Detailed information on the frequency of cancers during pregnancy is presented in Table 1 [8].

In this review, we aim to address issues regarding prognosis, diagnosis, treatment, and their consequences for both the mother and her offspring, with particular emphasis on considering the safety of the therapeutic and diagnostic methods used.

## 2. Materials and Methods

The following systemic review was conducted according to Preferred Reporting Items for Systemic Reviews and Meta-Analyses (PRISMA). The PubMed database was searched in order to find articles describing the latest information in line with the subject of this study, published between 2005 and 2023, with one study being older than this time period. The key words used in this research were melanoma AND pregnancy AND (fetus OR cancers in pregnancy OR diagnosis OR treatment).

### 2.1. Inclusion and Exclusion Criteria of the Study

Inclusion criteria for this study included the following: 1—studies that examined the epidemiology, diagnosis, and the safety and feasibility of treatment options for melanoma during pregnancy; 2—clinical trials; 3—randomized controlled trials; 4—systematic reviews; 5—meta-analyses; 6—reviews; and 7—case reports.

To guarantee that only top-tier primary evidence sources were included, the exclusion criteria included the following: 1—studies for which full text is not available; 2—studies that are not in the English language; 3—books and documents, book chapters, and conference papers; 4—unrelated studies; and 5—studies without sufficient data.

### 2.2. Results

Based on selected phrases with a time criterion applied, initially 580 items were searched in the PubMed database. In the first stage, based on the Free Full Text criterion, 230 items were removed, as well as 5 items due to being in a language other than English. After excluding books and documents, book chapters, and conference papers, 106 studies remained. Additionally, the searched phrases included therapeutic possibilities, safe diagnostics in pregnancy, and adverse effects of drugs for both mother and child, as well as therapeutic strategies for malignant tumors and the possibility of assessing the child’s well-being after treatment. After a thorough analysis of all criteria, 67 items remained, which were included in this review.

## 3. Prognosis

Reports on the prognosis of women with pregnancy-associated melanoma (PAM) are inconsistent. In some cases, an association between pregnancy and a poorer prognosis was confirmed [9,10], while other studies did not observe a significant impact on survival in women diagnosed with melanoma during pregnancy [11,12,13].

In a population-based study of pregnant women in California, 412 individuals with malignant melanoma diagnosed either during or within 1 year after pregnancy were identified. They were then compared with a control group of age-matched non-pregnant women who had also been diagnosed with melanoma. After adjusting for age, race, stage, and tumor thickness, it was found that pregnancy had no influence on the survival of women with melanoma. This remained consistent for both those diagnosed during pregnancy and those in the postpartum period [11].

The aim of another population-based cohort study was to assess mortality in women with PAM, diagnosed during pregnancy and up to 2 years postpartum. Among 6857 women diagnosed with cutaneous melanoma, 1019 cases were classified as PAM. The mortality rate in women with PAM did not differ from the mortality rate in women without PAM, providing no evidence of an adverse prognostic impact of pregnancy or recent childbirth [12].

In a study published in 2017, reproductive-age patients with cutaneous melanoma in stages 0 to IV were identified. The objective was to compare disease-free survival (DFS), overall survival (OS), and melanoma-specific survival (MSS) for women with PAM in stages 0-III compared to patients without PAM. In univariate or multivariate analysis, no significant differences were found in DFS, MSS, or OS for patients with PAM compared to patients without PAM, both in stages 0/I/II and stage III of cutaneous melanoma [13].

However, different results were obtained in a meta-analysis assessing the risk of death or recurrence in PAM compared to women of reproductive age with melanoma. The overall risk estimates for mortality showed an increased risk of death, after adjusting for patient age and melanoma stage, due to PAM compared to other forms of melanoma. Therefore, the conclusion was drawn that PAM has a poorer prognosis than other types of melanoma [9].

A retrospective study aimed at examining the histopathology, staging, risk factors, and course of cutaneous melanoma in women under 50 years old revealed that prognosis was significantly worse for patients with pregnancy-associated melanoma (PAM) compared to the control group of non-pregnant patients. There was a 9-fold increase in recurrence frequency, a 7-fold increase in metastases, and a 5-fold increase in mortality among patients with PAM [10].

## 4. Diagnosis

### 4.1. Physiological Changes during Pregnancy and Delayed Recognition

Despite the lack of significant evidence linking physiological changes in pregnant women to an increased risk of developing cancer, clinically significant aspects of pathophysiological changes during pregnancy can be outlined. These include the influence of sex hormone levels, primarily estrogens and progesterone, the impact of skin changes on the course of cancer diagnosis and delayed detection, fetal immunological tolerance, increased angiogenesis, and lymphangiogenesis, which may contribute to early metastases to sentinel lymph nodes [14,15,16,17,18,19].

Skin pigmentation is the most common symptom of pregnancy, occurring due to elevated levels of melanocorticotropin hormone (MSH), estrogen, and progesterone in the blood serum. Estrogen, reinforced by the action of progesterone, stimulates melanocytes to produce melanin. Changes are visible as early as the first trimester of pregnancy and occur especially in the areola and genital areas. Freckles, moles, or fresh scars, in addition to gradual darkening, may even enlarge. In the second trimester of pregnancy, the linea nigra becomes visible as a vertical line usually running from the pubic bone to the navel, although sometimes extending up to the chest. It typically fades a few months after childbirth. During pregnancy, pregnant women may also experience chloasma as a irregular, sharply defined brown pigmentation on the face, mainly in the cheek areas. Moreover, in the 6th to 7th month, over half of women may observe stretch marks on the abdomen, breasts, and thighs, appearing as reddish or bluish indentations [15]. Naturally occurring skin changes in pregnant women divert the attention of physicians, emphasizing the importance of their precise *examination*, considering changes over time. Any pigmentary change altering its clinical or dermoscopic character during pregnancy should be considered suspicious [14,16]. Vigilance in detection is crucial, as studies indicate that melanoma in pregnant women tends to have greater thickness than in women up to 12 months after delivery, and it occurs more frequently in first pregnancies than in subsequent ones [16].

Comparison was made between the development of angiogenesis and lymphangiogenesis in pregnant and non-pregnant women. The results revealed that the number of lymphatic vessels inside the tumor was significantly increased in pregnant women compared to non-pregnant women. In contrast, it was observed that melanoma progression is caused by angiogenic factors produced by the tumor, not by pregnancy [17].

There is evidence of the effectiveness of measuring local lymphangiogenesis in predicting the presence of metastases to lymph nodes. It can also be used to assess distant metastases. The method gains significance in predicting melanoma development in pregnant women, where the tendency toward metastases is increasing [14,18].

Studies have shown that despite the inflammation in the uterine mucosa during embryo implantation, an anti-inflammatory reaction occurs in the trophoblast after its formation, aiming at the immunological tolerance of the fetus and the maintenance of pregnancy [19]. Cancer cells can adopt many characteristics of the trophoblast to evade the immune system’s response. Additionally, the evolution of tolerance mechanisms to fetal neoantigens, especially those controlled by regulatory T lymphocytes, may play an important role in acquiring mechanisms that allow cancer cells to escape immune control. This immunological tolerance provides opportunities for tumors to progress and develop more rapidly since the body does not actively fight their presence [19,20,21].

Furthermore, the association between PAPP-A protein and accelerated melanoma progression has been investigated, particularly in the context of IGF1. PAPP-A exhibits strong expression in melanoma with metastases and serves as a prognosis for clinical outcomes. The inhibition of the protein using a neutralizing antibody or siRNA reduced the migration and invasion of melanoma cells in both in vitro and in vivo studies [22,23].

### 4.2. Observation of the Lesion and Dermatoscopy

The diagnostic process begins as soon as a concerning skin change, especially one rapidly enlarging, is observed. A family doctor or gynecologist can make such a diagnosis based on the ABCDE scheme (asymmetry, borders, color, diameter, and evolution of the lesion) and may refer the patient for further diagnostics [14,24,25].

It is recommended that for patients planning pregnancies with a history of melanoma, a Total Body Skin Examination (TBSE) be conducted lasting from 3 to 12 months, depending on the individual risk of recurrence and the emergence of new tumor-like changes. The last visit for such a patient is suggested to take place several months before delivery, in case staging, biopsy, or sentinel lymph node excision is required [24].

The dermoscopic diagnosis of nevi is based on the following four criteria (each characterized by four variables):

(a) color of the lesion;
(b) shape;
(c) pigmentation (multifocal, central, eccentric, and uniform);
(d) location, especially special sites—face, genital area, nails, and mucous membrane [25].

Moreover, the pattern of pigmentation of individual nevi may be influenced by six patient-related factors: age, skin type, a history of melanoma, UV radiation exposure, pregnancy, and growth dynamics [26].

Comparing the dermatoscopic features of nevi in the pregnant female population from the beginning of pregnancy, an increase in size, increased pigmentation, and an increase in their occurrence were observed in nearly half of the cases. The locations were mainly the abdominal and chest areas, rarely the arms or back. Importantly, these changes regressed or faded within a few months after delivery [25,27]. The increase in nevus size during pregnancy can be directly attributed to skin stretching and increased vascularity, which is confirmed in dermoscopic examination by pigmentary changes in the pigment network or vascular structures. However, nevi excised from pregnant patients may exhibit a benign degree of histological atypia or mitotic activity, which should not be overlooked or considered normal. Sometimes, the pathological examination of typical, benign nevi reveals the presence of skin mitoses, making the lesion actually resemble subtle nodular melanoma. To diagnostically address a skin change properly, knowledge of the patient’s history is crucial, taking into account any pregnancy or the period 3–6 months after delivery. If, despite a thorough clinical–pathological correlation and histological assessment, there are genuine doubts about whether the melanocytic lesion is benign or malignant, performing molecular studies on the lesion may be justified. This will provide additional evidence of the lesion’s malignant potential [25].

### 4.3. Staging

**Figure 1:**
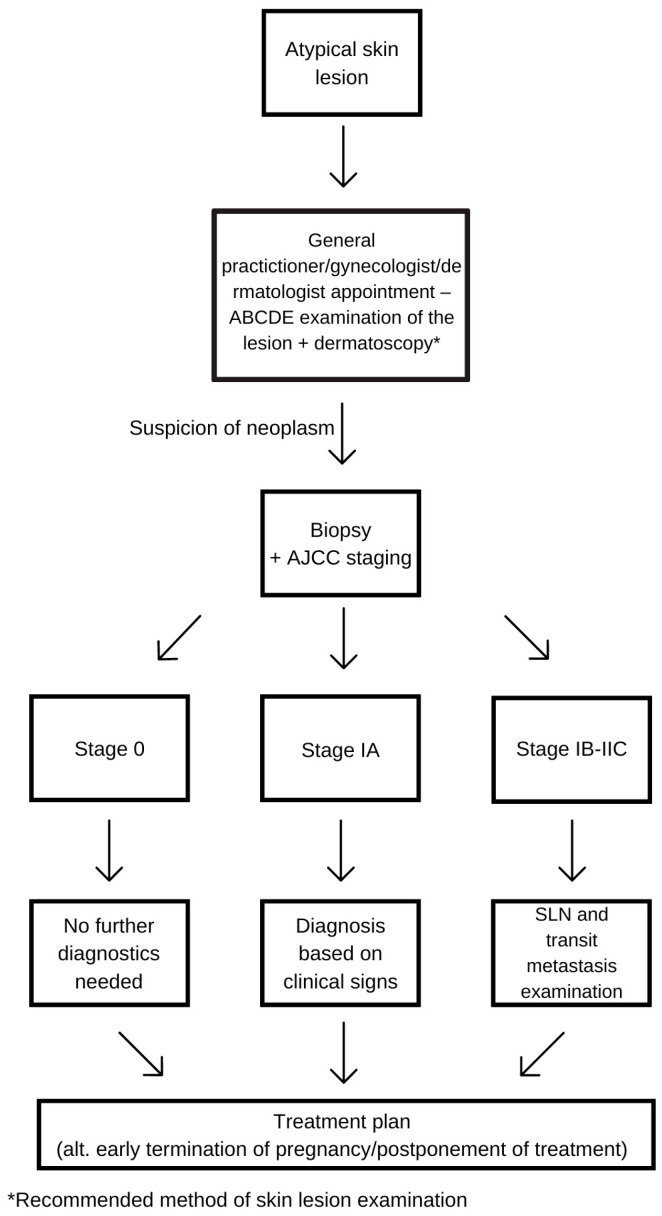
Diagnostic procedure diagram.

Regardless of pregnancy, any woman can safely undergo excisional or incisional biopsy if changes occur on the hands, soles, or facial skin. Lymph node biopsy and its wide local excision, performed under local anesthesia, are safe in any trimester of pregnancy, and their execution should not be delayed [14,22]. After the histological confirmation of melanoma, further diagnostic procedures depend on the stage of its development, the trimester of pregnancy, and the available safe tests. The tumor stage is determined according to the American Joint Committee on Cancer (AJCC). Stage 0 does not require further diagnostics. In stage IA, staging is based on clinical examination, while stages IB-IIC require checking for the involvement of regional lymph nodes and transit metastases. Only this ensures a correct classification into groups I/II or III. In stage IV, appropriate treatment for a pregnant woman is impossible. The patient must decide whether to delay treatment, terminate the pregnancy, or consider potential fetal risks associated with undergoing treatment [14,28] (Figure 1).

Various cases of pregnant women have been described in the literature, where the decision was made to excise rapidly growing lesions with a margin, under local anesthesia, typically turning out to be dysplastic pigmentary nevi [27]. However, a case has also been reported where the diagnosis was delayed and it was only initiated 2 years after the end of pregnancy, resulting in the detection of melanoma in stage IIIC. Unfortunately, metastatic changes in the brain and lungs appeared 6 months after tumor excision, ultimately leading to the patient’s death [29]. A swift diagnosis allows for the safe resolution of pregnancy and undergoing appropriate treatment without negative effects on the fetus, while ensuring good curability for the mother [30].

A negative impact on the prognosis in early postpartum detection significantly increases mortality in patients in the first period after childbirth. Pre-pregnancy diagnosis does not affect the prognosis. There is no evidence justifying the termination or postponement of pregnancy in patients diagnosed with melanoma [31].

However, the risk of death due to melanoma does not increase compared to non-pregnant women. No differences were found in the 10-year disease-free survival and overall survival rates between pregnant and non-pregnant groups. Previous pregnancies may have a favorable impact on the prognosis; in women who had had at least one pregnancy, there was a 94% chance of surviving melanoma compared to nulliparous women, of whom only 83% survived. A higher number of births and a younger age at first pregnancy were associated with reduced risk [32].

### 4.4. Sentinel Lymph Node Biopsy and Excision of the Lesion

Although recent studies have demonstrated the safety of mapping sentinel lymph node biopsy using Technetium-99, it is generally a matter of individual consideration of benefits and risks [33,34]. Radiation doses considered safe for the fetus are <100 mGy, and with Technetium, the dose is <5 mGy [22]. From a clinical perspective, there is significant concern about fetal anaphylaxis from blue dyes, particularly in the first trimester [14]. The risk of such an event is 1% [22]. The status of the sentinel lymph node is prognostically crucial for patients with a lesion of 1.0 mm. For diameters of 0.8–1.0 mm, an individual assessment should be considered [24]. However, lymphadenectomy under general anesthesia is risky, and it is advisable to wait until after pregnancy, especially as studies show no increased risk of a worse prognosis by waiting [24]. If the SLNB result is positive, the next appropriate step in diagnostics is imaging to determine the extent of the disease (National Comprehensive Cancer Network) [24,34].

The excision of melanoma during pregnancy is safe and diagnostically necessary. Tumors with a diameter of 1.01–2 mm should be excised with a margin of 1–2 cm according to the National Comprehensive Cancer Network. This procedure is performed under local anesthesia, similar to lymph node biopsy [24].

### 4.5. Radiology Diagnostic Possibilities

For pregnant patients, imaging methods involving ionizing radiation and radionuclides should be limited. According to the American College of Obstetricians and Gynecologists Practice Committee, preferred imaging techniques during pregnancy include chest X-rays with appropriate shielding, ultrasonography, and magnetic resonance imaging. The use of gadolinium contrast in MRI is not recommended. If necessary, non-contrast CT and nuclear medicine studies can be performed since they are usually administered in doses that have not shown fetal harm [24,34,35]. PET/CT scans covering the entire body are likely the best imaging method for detecting melanoma metastases, potentially altering the treatment plan in nearly 50% of cases [36].

It should be remembered that in diagnostic radiology, the most sensitive period for organogenesis is between the 18th and 38th day of pregnancy [37]. Exposure to radiation in the second or third trimester is associated with the development of intellectual impairment, developmental defects in the central nervous system or gonads, and premature death. With various imaging procedures, average doses to the uterus/fetus range from 0.001 mGy for chest X-rays to 25 mGy for abdominal and pelvic computed tomography [38]. Ultrasound (USG) and magnetic resonance imaging (MRI) are considered safe during pregnancy as they use non-ionizing radiation. Although there are theoretical risks associated with acoustic damage and fetal heating during MRI, no increased risk of vision loss, hearing loss, fetal necrosis, or congenital defects related to MRI has been observed in the first trimester. Nevertheless, the use of gadolinium-based contrast increases the risk of developing rheumatologic, inflammatory, or infiltrative skin and kidney diseases, as well as fetal necrosis and newborn death. The use of gadolinium should be limited to situations where benefits outweigh risks [37,39]. Because conventional X-ray radiation, computed tomography, and nuclear medicine studies involve ionizing radiation, the gestational age and fetal dose should be considered when using these imaging methods. The Imaging Wisely initiative, a joint effort of the American College of Radiology, the Radiological Society of North America, the American Society of Radiological Technologists, and the American Association of Physicists in Medicine, recommends that if positron emission tomography (PET) is indicated during pregnancy, a reduction in FDG dose to 5 mCi should be applied to minimize fetal exposure [37].

### 4.6. New Diagnostic Possibilities

Video-assisted inguinal lymphadenectomy (VIL) is a minimally invasive alternative associated with fewer complications and shorter hospital stays. A case has been described where VIL was successfully performed on a pregnant mother in the 24th week of pregnancy with a history of melanoma and a current recurrence. The package of cancer-involved lymph nodes was removed. This method allows a safe approach to melanoma diagnostics without delaying aggressive cancer treatment [40].

SIAscopy, defined as Spectrophotometric Intracutaneous Analysis, is a new non-invasive skin imaging technique that describes the pattern of melanin distribution in the epidermis and the papillary layer of the skin, total and epidermal melanin, collagen pattern, and blood perfusion. SIAscopy is a unique skin imaging technology that uses visible and infrared light in combination with an advanced model of light transport physics in the skin to create subsurface maps of various chromophores, including hemoglobin, melanin, and collagen. Spectrophotometric images describe the live microarchitecture of pigmented lesions up to a depth of 2 mm below the skin surface. All information is stored in databases, allowing easy access and the comparative analysis of these changes over time. SIAscopy appears to be an innovative method in the diagnosis of melanoma in pregnant women [41].

## 5. Diagnostics and Treatment

The treatment of cancer during pregnancy usually obliges clinicians to modify standard therapies, choose less invasive treatments, or harmful drugs, or even sometimes postpone treatment until after the postpartum. The aforementioned adjustments are primarily determined by factors directly related to possible harmful, teratogenic, or even lethal effects on the developing fetus, as well as by changes in the mother’s body, which occur during pregnancy, concerning multiple systems, such as the cardiovascular, endocrine, immune, and many other systems. Limited diagnostic capabilities (as described above) are likely to complicate further therapeutic decisions, so that the severity of the disease and the presence of possible metastases may be underestimated or overestimated, leading to the selection of inadequate treatment methods [42].

### 5.1. Surgery of the Primary Melanoma

The most effective and common way to treat primary melanoma is to excise the lesion as soon as possible after diagnosis, with an adequate margin (both laterally and in depth) determined by the results of the histopathological examination of the biopsy material [34,43,44,45]. This crucial procedure should not be delayed, although the second trimester is the most favorable and recommended time of conducting the procedure [34]. Since the cancer occupies the skin, surgical access is usually safe and unobstructed, and thus the procedure itself can be performed under local anesthesia only [34,43,44,45]. Preference is given to the use of lidocaine which belongs to FDA category B, that despite its transmission across the placenta, has no negative effects on the fetus. Moreover, its side effect profile mainly includes harmless tachycardia and dizziness in pregnant women [46]. In addition, the use of epinephrine can be considered as an agent that prolongs the effect of the local anesthetic and allows a reduction in its used dose [34]. Although the drug is classified as FDA category C, there are only a few documented cases of malformations manifested after exposure to high doses of epinephrine [34,46]. Furthermore, the doses used in local anesthesia are up to 20 times lower than the endogenous release of adrenaline in a stressful situation for the organism [34]. Therefore, possible side effects that the drug could cause after passing through the placenta, such as decreased placental perfusion and fetal tachycardia, are extremely rare [46]. The aforementioned medical evidence determines that the combination of low-dose lidocaine and epinephrine not only provides effective but also safe anesthesia in pregnant women, which makes it generally recommended and widely used [47]. However, the use of drugs, such as mepivacaine and bupivacaine (FDA category C), which may cause bradycardia and malformations in the fetus, is strictly contraindicated [34].

### 5.2. Metastases and Recurrence of Melanoma

Malignant melanoma can give distant metastases and characteristic transit, as well as satellite metastases, which require the usage of appropriate treatment methods [48].

Satellite metastases, that is, those that occupy the skin around the area of the primary lesion, are supposed to be excised along with it [44].

Transit metastases to sentinel lymph nodes (SLNs) cause more of a problem because even if they are accessible and surgically removable, general anesthesia of the patient is necessary [21]. Adaptive changes in the maternal organism during pregnancy, such as hypotension, an increased risk of bleeding, hypercoagulability, tachycardia, and decreased lower esophageal sphincter (LES) activity, are very likely to complicate the course of further anesthesia [49]. Although necessary, general anesthesia unfortunately has an inherent risk of both maternal and fetal complications [49,50,51]. Until now, the “optimal” choice of anesthetics has not been established, because almost all of the drugs used in GA (general anesthesia) pass through the placenta and belong to FDA category C, so they can induce teratogenic effects [50]. In addition, the disturbance of normal maternal–fetal circulation, due to hypotension associated with GA, can cause asphyxia in the fetus [49,51]. The combination of all the factors relevant to the mother and child during GA can lead to preterm labor and even result in fetal death in about 5.8% of cases [49].

Distant metastases and inaccessible locations of the tumor on the skin can make melanoma unresectable, that is, impossible to remove completely during surgical operation. In such situations, alternative treatment such as radiotherapy and immunotherapy may be necessary. Unfortunately, it cannot be frequently used in the treatment of PAM, contrary to melanoma outside of pregnancy [22,43,44,45].

### 5.3. Radiation

The potential use of radiotherapy in the treatment of PAM should be carefully considered for a variety of reasons. Usually, the single radiographic tests taken for diagnostic purposes, even a single-phase CT scan of the abdomen or pelvis, deliver less than 50 mGy to a fetus and are harmless. However, the basic principle of radiological protection, called ALARA (As Low As Reasonably Achievable) underlines that there is no safe threshold for the stochastic effect of ionizing radiation. Therefore, all possible actions to reduce the dose of radiation should be considered. Ionizing radiation can cause direct damage to DNA, including single- or double-strand breaks and loss or damage to the bases and indirect changes, which are the result of the ionization of intracellular water and the appearance of highly reactive chemical species, like hydroxyl or hydrogen radicals and solvated or aqueous electrons. These changes can be either lethal or sublethal, meaning that they can lead to an abnormal function of the cell, but there is also the possibility of successful repair. The damage especially affects less differentiated and actively proliferating cells. That is the reason why the tissues of a human fetus are most vulnerable to radiation [52]. Possible risks of radiation to the conceptus depend on the gestational age and the dose. For instance, doses higher than 100 mGy can cause spontaneous abortion in the first two weeks of pregnancy, possible malformations in the first trimester, and the risk of mental retardation in the second trimester [53]. Fetal exposure even to a low dose of radiation can also be associated with childhood and adult cancer risk [54].

Complications of higher dose imaging included fetal malformations, microcephaly, mental retardation, growth restriction, a higher risk of malignant neoplasm (mostly leukemias), and even pregnancy loss. In the preimplantation period, there was a risk of embryo implantation failure [19,22,55,56].

In addition, the effectiveness of this method is lower than systemic chemo/immunotherapy, which also influences the decision to use it less often [43,44]. The advantage of radiation therapy over surgery remains a better cosmetic effect, so it can be considered, for example, when cancer occupies the skin of the face, or other hard-to-reach areas, when safer and more effective alternative methods are lacking [44].

### 5.4. MRI

Currently there is no evidence that MRI is not safe for the fetus; nevertheless, not enough studies have been carried out and our knowledge is still limited. Due to unidentified biological results of high magnetic fields in the period of organogenesis, experts suggest performing MRI after 18 weeks of pregnancy. The biological response of the cells to MRI causes the heating of tissues, which can be potentially destructive for developing organs [57]. Because of uncertainties about the impact of MRI on the fetal growth and the damage to the inner ear due to high acoustic disturbances, some recent studies have been performed. They showed no links between exposure to the procedure of 1.5 T MRI during pregnancy and the disfunction of the inner ear of a child [58]. However, the use of gadolinium for pregnant women still remains controversial as its safe administration has not been proven. The MRI procedure in pregnant women is generally performed without gadolinium-enhanced images, due to potential side effects to the fetus [57,58].

A two-arm cohort study examined the potential risk of MRI between 2 and 14 weeks of gestation or gadolinium-enhanced MRI between 2 weeks of gestation and 2 days before scheduled delivery on the developing fetus. Although death from congenital malformations, hearing loss, or developing a neoplasm was not more frequent in the trial with or without gadolinium, there was an increased risk of neonatal death, stillbirth and the occurrence of rheumatological, infiltrative, and inflammatory skin diseases when gadolinium was implemented, especially in procedures performed during the first trimester. So far, there are no studies confirming concerns that MRI with a higher magnetic field (3T) may be ototoxic to the developing fetus [14,39,56].

### 5.5. Systemic Therapy

Currently, the standard of care for stage IIB to IV melanoma is adjuvant immunotherapy (checkpoint immunotherapy) or BRAFV600 mutation-targeted therapy (braf-targeted and MEK-targeted), which, when used postoperatively, reduce the risk of recurrence and extend distant metastasis-free survival. Neoadjuvant immunotherapy also has clinically proven efficacy in reducing tumor mass before surgery, but has not yet been introduced into routine care [43]. Difficulties in the potential introduction of immunotherapy into the treatment of PAM currently arise from the inability to conduct proper clinical trials. Although they could clearly determine the therapeutic potential of this method, for obvious ethical reasons, they cannot be conducted on pregnant patients. Relatedly, the source of current knowledge on the use of PD-1, LAG-3, BRAF kinases, and MEK inhibitors are preclinical studies and individual case reports, in which patients have chosen to continue treatment during pregnancy or have become pregnant during therapy treatment [14,59,60,61].

PD-L1 and CTLA-4 blockage by using pembrolizumab, ipilimumab, and nivolumab showed an increased risk of miscarriage in animal studies. Nivolumab also caused fetal abnormalities and increased neonatal mortality but there was no such complication during pembrolizumab therapy [19,62]. An increased probability of stillbirth, preterm labor, and neonatal death during ipilimumab treatment was proven in monkey studies [63]. Despite the immediate discontinuation of therapy with nivolumab, complications such as spontaneous preterm birth, moderate intrauterine growth restriction, and congenital hypothyroidism occurred [60]. A recent study conducted on seven obstetric patients with melanoma showed complications such as premature birth, intrauterine growth retardation, and HELLP syndrome. Reassuringly, no infant had long-term complications [14]. Immunotherapy could cause neonatal endocrine disorders such as congenital hypothyroidism, affecting fertility [35].

A similar risk was posed using BRAF/MEK inhibitors on animal fetuses where the risk of miscarriage or embryonic malformations was higher [62]. Dabrafenib was proven to have both teratogenic and embryotoxic influence, and encorafenib may cause fetal damage. Of four cases with vemurafenib medication, all neonates were born premature and one of them also had growth restriction, although none of the complications were introduced during studies in animals. We can conclude that combined BRAF and MEK inhibitor treatment is not a reliable option [14,63].

Complementary treatment is also abandoned to reduce the potential risk of complications for the fetus [14].

Even up to 30% of patients treated with ICI in combined therapy can have autoimmune endocrine side effects such as hypothyroidism, hyperthyroidism, Addison’s disease, hypophysitis, and type 1 diabetes. Many of them can lead to severe complications and also affect fertility. Fortunately, a great number of repercussions can be reduced using hormone replacement therapy [62].

**Figure 2:**
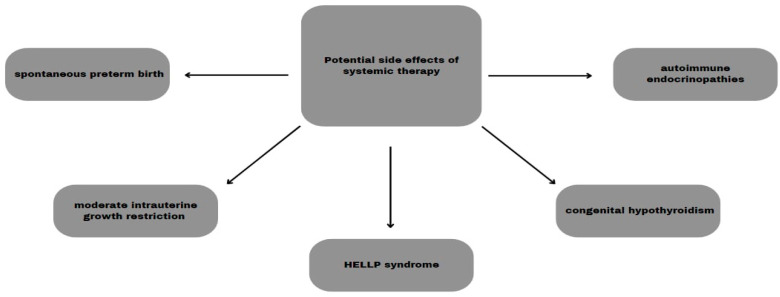
Potential side effects of systemic therapy.

The potential side effects of systemic therapy are summed up in Figure 2.

Chemotherapy with ‘classic’ cytotoxic anticancer drugs, using, for example, temozolomide or dacarbazine, is not currently used in the treatment of melanoma due to unsatisfactory efficacy relative to increased toxicity and the risk of miscarriage [64].

Available treatment methods which can be used in PAM are summarized in Table 2.

## 6. Consequences for Both Mother and Child

Studies assessing the impact of melanoma treatment on fetuses were performed mostly on animals. Therefore, the actual impact of most of them on human pregnancies is unclear, and some of the results are not clinically relevant. Anesthetics, antibiotics, and imaging and invasive procedures can cause a number of complications. This results in a general recommendation that all patients use contraception during treatment and up to 5 months. Moreover, it is strongly advised to use the dual contraceptive during ICI therapy, because its gastrointestinal side effects can reduce the effectiveness of oral hormonal contraception [14,34,62].

### 6.1. Perinatal Complications

Among examined groups, the frequency of preterm labor was 8.7% and that of low birth weight was 5.1%. In comparison, the percentages in the general population were 10% and 8%. The risk ratios of melanoma were 0.9 for labor >37 weeks of gestation and a birth weight below 2500 g, and 0.8 for small gestational age. In conclusion, no association was found between the three listed factors and the incidence of melanoma diagnosed during pregnancy. Moreover, among pregnancy-associated neoplasms, melanoma had a low stillbirth rate of 15%. On the other hand, an increased risk of too large neonates for their gestational age was noticed [22,65,66].

### 6.2. Placental Metastasis

In rare cases, the cancer may spread to the fetus via the transplacental route. Melanoma is the neoplasm with the highest rate of placental metastasis [67]. All cases are accompanied by cancer dissemination to the mother’s internal organs [14]. The risk of placental tumors being metastasized to the fetus is estimated to be about 17% [19]. We can assume that most of the pregnancies have a good outcome, although if we detect neoplasm in the fetus, treatment is usually ineffective and leads to an infant mortality rate of over 80%. That is why in all patients with stage IV melanoma, the placenta should be diligently examined histologically [14]. One multicenter retrospective study revealed that in a group of 63 live-born children, none of them had evidence of fetal or placental metastases. In the comparison group, 14 cases with fetal metastases were identified. Ten of them (71.43%) resulted in the death of the offspring, preceded by the metastatic spread mainly to the lungs, liver, and brain on average 4.33 months after birth. Mean survival time after diagnosis ranged from 2 weeks to 28 months [67]. Moreover, healthy neonates with a medical history of placental metastases are at high risk and must be monitored every 3 months for the first 24 months of life [35,67]. A detailed review of metastatic melanoma in pregnancy and the risk of transplacental metastases in the infant was performed by Alexander et al. [68].

### 6.3. Maternal Organism

There is no evidence to conclude that melanoma diagnosed during pregnancy is associated with a poorer prognosis. However, some studies report higher mortality in obstetric patients by 17%. More research is needed to determine the risk [14,22,35].

## 7. Conclusions

Pregnancy-associated melanoma, despite being a statistically rare disease, poses a significant and complex problem for the patient as well as clinicians from different specializations. There are certain factors that can increase the risk of melanoma development in pregnant women, such as hormonal changes, an excessive exposure to UV radiation before or during pregnancy, and immunological changes occurring in the woman’s body during pregnancy. The diagnosis of melanoma in pregnant women should be individualized and requires the cooperation of an interdisciplinary treatment team, including dermatologists, oncologists, gynecologists, and neonatologists. It is extremely important for pathologists to conduct a thorough examination of the placenta and the child after birth to grossly and histologically detect any possible melanoma metastases. During the treatment of pregnancy-associated melanoma (PAM), it is necessary to take all fetal and maternal factors into consideration and adjust the therapy accordingly to make it effective and safe for both mother and child.

It is important for pregnant women to regularly check their skin for changes, such as new moles or moles changing in shape, color, or size, and report any concerning changes to their doctor. The early detection and proper treatment of melanoma increase the chances of successful recovery.

## Figures and Tables

**Table 1:** The frequence of cancers during pregnancy.

Cancer Type	%
Breast Cancer	41%
Lymphoma	12%
Cervical Cancer	10%
Leukemia	8%
Ovarian Cancer	7%
Gastrointestinal Cancer	5%
Melanoma	5%
Other	12%

**Table 2:** Treatment methods in PAM.

Melanoma Staging	Recommended Therapy
Stage 0 (Melanoma in situ)	Excision of the lesion ^a^
Stage I	Excision of the lesion ^a^ + SLNB
Stage II	Excision of the lesion ^a^ + SLNB
Stage III (lymph nodes involved)	Excision of the lesion and sentinel/involved lymph nodes ^b^; Consider preponed labor; Adjuvant immunotherapy ^c^
Stage IV (presence of distant metastases)	Treatment based on clinical presentation and advancement of the pregnancy ^d^; Excision of the lesion and sentinel/involved lymph nodes ^b^; Consider preponed labor; Adjuvant immunotherapy ^c^ Radiotherapy ^e^

^a^—under local anesthesia; ^b^—under general anesthesia; ^c^—after postpartum; ^d^—all possible negative effects on the child must be considered; ^e^—in supradiaphragmatic localization; SLNB—sentinel lymph node biopsy; if lymph nodes are involved, staging immediately changes to stage III.

## Data Availability

The data presented in this study are available in this article.
